# Fine-scale population epigenetic structure in relation to gastrointestinal parasite load in red grouse (*Lagopus lagopus scotica*)

**DOI:** 10.1111/mec.12833

**Published:** 2014-07-24

**Authors:** MARIUS A WENZEL, STUART B PIERTNEY

**Affiliations:** Institute of Biological and Environmental Sciences, University of AberdeenZoology Building, Tillydrone Avenue, Aberdeen, AB24 2TZ, UK

**Keywords:** DNA methylation, epigenetics, genotype-environment association, host-parasite interactions, induced phenotypes, methylation-sensitive AFLP

## Abstract

Epigenetic modification of cytosine methylation states can be elicited by environmental stresses and may be a key process affecting phenotypic plasticity and adaptation. Parasites are potent stressors with profound physiological and ecological effects on their host, but there is little understanding in how parasites may influence host methylation states. Here, we estimate epigenetic diversity and differentiation among 21 populations of red grouse (*Lagopus lagopus scotica*) in north-east Scotland and test for association of gastrointestinal parasite load (caecal nematode *Trichostrongylus tenuis*) with hepatic genome-wide and locus-specific methylation states. Following methylation-sensitive AFLP (MSAP), 129 bands, representing 73 methylation-susceptible and 56 nonmethylated epiloci, were scored across 234 individuals. The populations differed significantly in genome-wide methylation levels and were also significantly epigenetically (*F*_SC_ = 0.0227; *P* < 0.001) and genetically (*F*_SC_ = 0.0058; *P* < 0.001) differentiated. Parasite load was not associated with either genome-wide methylation levels or epigenetic differentiation. Instead, we found eight disproportionately differentiated epilocus-specific methylation states (*F*_ST_ outliers) using bayescan software and significant positive and negative association of 35 methylation states with parasite load from bespoke generalized estimating equations (GEE), simple logistic regression (sam) and Bayesian environmental analysis (bayenv2). Following Sanger sequencing, genome mapping and geneontology (go) annotation, some of these epiloci were linked to genes involved in regulation of cell cycle, signalling, metabolism, immune system and notably rRNA methylation, histone acetylation and small RNAs. These findings demonstrate an epigenetic signature of parasite load in populations of a wild bird and suggest intriguing physiological effects of parasite-associated cytosine methylation.

## Introduction

The traditional paradigm that phenotypic variability and evolutionary change are consequences solely of DNA sequence variation is becoming increasingly challenged (Jablonka & Lamb 2007; Bonduriansky & Day 2008; Danchin *et al.* 2011; Mesoudi *et al.* 2013). The emerging field of epigenetics is concerned with dynamic, yet mitotically and sometimes meiotically stable, regulatory patterns of gene expression and chromatin remodelling in the absence of nucleotide sequence variation (Jablonka & Raz 2009; Massicotte *et al.* 2011; Alabert & Groth 2012). From a molecular ecology perspective, these phenomena complement the DNA sequence-based systems hitherto examined and are likely to provide new insights into the underpinning, regulation and evolution of phenotypic traits (Bossdorf *et al.* 2008; Angers *et al.* 2010; Richards *et al.* 2010; Duncan *et al.* 2014).

The most intensively studied epigenetic mechanism is enzymatically mediated attachment of a methyl group to cytosine or adenine nucleotides (Angers *et al.* 2010). Such DNA methylation is taxonomically widespread, but its extent and function are highly taxon specific (Suzuki & Bird 2008; Angers *et al.* 2010; Jones 2012). In plants, for example, cytosine in any trinucleotide (CpNpN) may be methylated, whereas in vertebrates, methylation is almost exclusively limited to cytosine in CpG dinucleotide sites (Angers *et al.* 2010; Fulneček & Kovařík 2014). Cytosine methylation may display a number of different effects depending on functional sequence context. Increased methylation of CpG islands in gene promoters is often associated with a decrease in the expression of those genes (Angers *et al.* 2010; Jones 2012; Duncan *et al.* 2014). In contrast, methylation in gene bodies or noncoding regions may, for example, silence transposable elements or genomic parasitic sequences (Suzuki & Bird 2008; Zemach *et al.* 2010; Jones 2012), provide mutational hot spots through increased deamination rate of methylated cytosine (Lutsenko & Bhagwat 1999; Poole *et al.* 2003; Jones 2012), or recruit protein complexes and factors that are involved in chromatin remodelling (Jaenisch & Bird 2003; Bannister & Kouzarides 2011).

Mitotic stability of methylation patterns during ontogenesis is a key mechanism that not only mediates cell differentiation, but in concert with malleability of methylation states also provides a framework for environmental factors to influence phenotype expression during early developmental stages (Bird 2002; Skinner 2011; Feil & Fraga 2012; D’Urso & Brickner 2014). Moreover, compelling evidence is accumulating for environmentally induced changes in methylation patterns long after ontogenesis (Duncan *et al.* 2014). Not only may such changes underpin phenotypic plasticity during an individual's lifetime (Jaenisch & Bird 2003; Bossdorf *et al.* 2008; Angers *et al.* 2010; Stevenson & Prendergast 2013; Duncan *et al.* 2014), but some of these patterns may also be vertically transmitted, either directly through meiotic stability of methylation patterns or indirectly by transmission of extragenomic molecules in gametes (Jablonka & Raz 2009; Petronis 2010; Skinner 2011; Smith & Ritchie 2013; Duncan *et al.* 2014). Recent studies highlight a role for methylation in broad eco-evolutionary processes such as biological invasion (Richards *et al.* 2012), sexual selection (Crews *et al.* 2007), domestication (Xiang *et al.* 2013), inbreeding depression (Vergeer *et al.* 2012), seasonal timing of physiology (Stevenson & Prendergast 2013), transition between maturation stages (Morán & Pérez-Figueroa 2011) and reproductive labour division in social insects (Amarasinghe *et al.* 2014). On a population epigenetics level, differentiation of methylation states is frequently observed among populations in different environments (Herrera & Bazaga 2010; Lira-Medeiros *et al.* 2010; Liu *et al.* 2012; Schulz *et al.* 2013). Such epigenetic differentiation has also been demonstrated to be meiotically persistent (Salmon *et al.* 2008; Herrera *et al.*, 2013,^2014^), implying a potential role for local adaptation and speciation (Bossdorf *et al.* 2008; Roux *et al.* 2011; Richards *et al.* 2012; Smith & Ritchie 2013).

Particularly useful insights on the mechanistic contribution of epigenetics to plasticity and adaptation come from exploring the epigenetic effects of particular environmental stresses (Feil & Fraga 2012). For example, osmotic stress caused by transition from fresh water to sea water induces methylation-mediated acclimation processes in brown trout (Morán *et al*. 2013). Similarly, methylation-mediated nutritional plasticity as a result of changes in the nutritional environment has been found in a nectar-eating yeast (Herrera *et al.* 2012) and in horned beetle larvae (Snell-Rood *et al.* 2013). Numerous studies on plants have identified methylation effects of various abiotic stressors, for example temperature (Paun *et al.* 2010), nutrient availability (Verhoeven *et al.* 2010), water availability (Paun *et al.* 2010) and osmotic stress (Chinnusamy & Zhu 2009; Tan 2010). Compelling evidence for methylation responses to biotic factors, such as pathogens and herbivory, also comes from plant studies (Herrera & Bazaga 2011; Dowen *et al.* 2012), where even the experimental application of damage-associated plant hormones elicits heritable methylation changes associated with a concomitant stress response (Verhoeven *et al.* 2010). Parasites are extremely potent stressors with profound eco-evolutionary importance (Bérénos *et al*. 2011; Gómez-Díaz *et al*. 2012; Mostowy & Engelstädter 2012), yet little is known about parasite-associated epigenetic effects in animal hosts. Notably, helminth parasites have been linked to carcinogenesis (Fried *et al.* 2011), most prominently in bladder cancer, where patients with schistosome infection have consistently different tumoral methylation patterns compared with noninfected patients (Gutiérrez *et al*. 2004). Considering the large gamut of physiological and evolutionary consequences of parasite infection, studying host–parasite systems in an ecological epigenetics context promises to be an exciting, yet challenging, avenue of research (Poulin & Thomas 2008; Gómez-Díaz *et al*. 2012; Biron & Loxdale 2013; Poulin 2013).

An extremely well-studied natural host–parasite system that is well suited for exploring ecological epigenetics is the red grouse (*Lagopus lagopus scotica*) and its gastrointestinal nematode parasite *Trichostrongylus tenuis*. Red grouse are endemic to the heather moorlands of upland Scotland and Northern England, where their environment is intensively managed for sport shooting purposes (Martínez-Padilla *et al.* 2014). Male grouse are highly philopatric and territorial, particularly in autumn when elevated testosterone enhances aggression among young cocks and their kin during recruitment. Young cocks attempt to establish a territory in the immediate vicinity of their kin, resulting in spatial kin structures within populations (Watson *et al.* 1994; Piertney *et al.* 1999, 2000; MacColl *et al.* 2000) and also some degree of genetic structure among populations (Piertney *et al.* 1998, 2000). *T. tenuis* exhibits a direct life cycle and is a major driver of red grouse ecology (Martínez-Padilla *et al.* 2014). Infectious larvae are ingested with heather shoots and establish in the caecum where adult parasites cause haemorrhaging that results in poor physiological condition and compromised survival and fecundity (Watson *et al.* 1987; Hudson *et al.* 1992; Delahay *et al.* 1995; Delahay & Moss 1996). At least 90% of grouse in a population are infected (Wilson 1983) and, although a specific immune response is mounted (Webster *et al.* 2011a), grouse typically cannot acquire full immunity and therefore continue to bear specific parasite burdens that vary considerably among individuals (Shaw & Moss 1989).

Chronic parasite infection has marked effects on grouse behaviour and physiology. High parasite load reduces territorial aggression in male grouse (Fox & Hudson 2001; Mougeot *et al.* 2005a), which has knock-on effects on recruitment and kin structure (Moss & Watson 1991; Mougeot *et al.* 2005b) and may ultimately contribute to the instability of grouse population dynamics (Hudson *et al.* 1998; Redpath *et al.* 2006; Martínez-Padilla *et al.* 2014). Moreover, parasite infection has a range of physiological consequences that underpin sexual selection processes in grouse populations (e.g. Mougeot *et al.* 2004; Seivwright *et al.* 2005; Martínez-Padilla *et al.* 2007, 2010; Vergara *et al.* 2012). Both male and female grouse possess carotenoid-based supra-orbital combs that function in males as testosterone-dependent signals of condition. Parasite load is intricately linked to various components of condition, such as immune function (Mougeot *et al.* 2004; Mougeot & Redpath 2004; Mougeot 2008), oxidative status (Mougeot *et al.* 2009, 2010a) or physiological stress (Bortolotti *et al.* 2009; Mougeot *et al.* 2010b), suggesting a key role in signal modulation. Indeed, parasite infection not only elicits transcriptomic up-regulation of immune system processes and stress responses (Webster *et al.* 2011a, b), but also interacts with testosterone to depresses immunity and oxidative damage responses consistent with transcriptomically mediated handicap mechanisms (Wenzel *et al.* 2013). Taken together, these studies highlight a key role of parasites to alter physiological processes in red grouse and suggest changes in gene expression as an important mechanism. Such gene expression changes could potentially be regulated or modulated by epigenetic mechanisms such as cytosine methylation, through, for example, changes in gene promoter methylation or chromatin remodelling (Bird 2002; Jaenisch & Bird 2003; Angers *et al.* 2010; Jones 2012). More generally, parasite-associated changes in methylation patterns in the absence of genetic variation could act as a regulatory component of physiological stress responses to parasite infection (Poulin & Thomas 2008; Gómez-Díaz *et al*. 2012).

Paramount to approaching these intriguing ideas in an ecological context is exploring correlational epigenetic signatures of parasite load in natural populations. Here, we present an ecological epigenetics study on parasite-associated genome-wide cytosine methylation patterns in a landscape system of red grouse populations at high autumnal testosterone levels in north-east Scotland. Our objectives are threefold: First, we epigenotype individuals at genome-wide anonymous CpG sites using methylation-sensitive AFLP (MSAP) to estimate methylation levels and patterns of epigenetic differentiation among populations. Second, we test for associations of the identified genome-wide and locus-specific methylation patterns with parasite load. Finally, we sequence those identified loci to ascertain the potential physiological effects of parasite-associated methylation changes. Our results highlight significant epigenetic differentiation among the sampled grouse populations and significant association of parasite load with locus-specific methylation states, but not genome-wide methylation levels or spatial epigenetic structure.

## Materials and methods

### Sample collection

**Fig 1:**
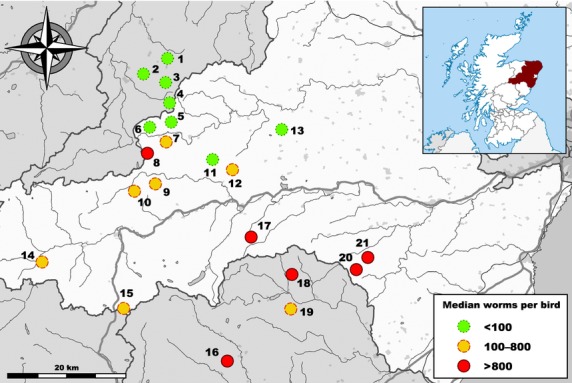
Sites in Aberdeenshire, Angus and Moray that were sampled following grouse shoots in autumn 2012. Detailed locations, sample sizes and parasite loads are given in Table1.

A total of 234 shot grouse were sampled in autumn 2012 following driven or walked-up sporting shoots at 21 sites near Deeside, Aberdeenshire (Fig.1, Table1). Age was determined as ‘young’ (<1 year) or ‘old’ (>1 year), and, where possible, only old birds were sampled in order to minimize bias by sampling of kin groups. Weight was measured to the nearest 10 g with a spring balance, and supra-orbital comb size (width and length) was measured to the nearest mm. Caecal content samples were taken for parasite load estimation following faecal parasite egg counts (standard McMaster chamber slide method; Seivwright *et al.* 2004). Liver samples were taken for DNA extraction because liver is a homoeostatically and immunologically important organ that is well suited to explore systemic parasite-specific effects (Racanelli & Rehermann 2006; Webster *et al.* 2011a) and typically yields large amounts of contaminant-free high-quality DNA necessary for restriction-based assays (Benjak *et al.* 2006; Wong *et al.* 2012). DNA was extracted from 2 to 3 c. 2 mm^3^ shreds of liver tissue according to Hogan *et al*. (2008). DNA quality and quantity were assessed with a NanoDrop ND-1000 spectrophotometer, and extracts were diluted to c. 100 ng/μL. PCR-based sex determination using a gonosome-linked locus (Griffiths *et al.* 1998) was performed as described in Wenzel *et al*. (2012).

**Table 1:** Sampling locations, sample sizes (M = male, F = female, Y = young) and parasite loads (median worms per bird with 25% and 75% quantiles)

Sampling locations				Sample sizes				Worms per bird		
Site	Estate	Long.	Lat.	Total	M	F	Y	25%	Median	75%
1	Glenlivet	57.29	−3.18	10	4	6	0	4	4	632
2	Glenlivet	57.25	−3.28	10	7	3	0	4	4	4
3	Edinglassie	57.24	−3.20	10	6	4	0	4	4	4
4	Edinglassie	57.21	−3.19	10	7	3	0	4	4	4
5	Allargue	57.19	−3.29	10	4	6	0	4	4	4
6	Allargue	57.19	−3.23	10	6	4	10	4	4	4
7	Delnadamph	57.16	−3.26	10	5	5	0	380	582	1394
8	Delnadamph	57.14	−3.30	11	9	2	0	947	1215	1715
9	Invercauld	57.10	−3.29	10	3	7	5	4	513	1586
10	Invercauld	57.08	−3.35	10	5	5	5	150	500	2264
11	Dinnet	57.12	−3.11	10	8	2	0	4	40	112
12	Dinnet	57.11	−3.06	10	6	4	0	4	180	556
13	Tillypronie	57.18	−2.94	9	3	6	8	4	78	200
14	Mar Lodge	56.95	−3.66	11	6	5	3	315	676	1400
15	Invercauld	56.87	−3.40	15	13	2	0	222	602	800
16	Airlie	56.81	−3.08	18	13	5	0	812	2222	4069
17	Glen Muick	56.99	−3.01	20	11	9	0	674	1586	2609
18	Invermark	56.94	−2.89	10	6	4	0	600	1084	1380
19	Invermark	56.89	−2.89	10	4	6	0	232	603	694
20	Glen Dye	56.95	−2.72	10	6	4	5	372	813	1141
21	Glen Dye	56.96	−2.69	10	6	4	5	358	1006	1566
				234	138	96	41			

### Methylation-sensitive AFLP

Methylation-sensitive AFLP (Reyna-López *et al*. 1997) allows for assaying methylation at anonymous 5’-CCGG restriction sites in a similar fashion to classic AFLP assays of genetic variation (Vos *et al.* 1995). Genomic DNA is restricted in two parallel digests per sample, each containing *Eco*RI and one of the two isoschizomers *Msp*I and *Hpa*II that differ in sensitivity to cytosine methylation configurations of the 5’-CCGG restriction site (Herrera & Bazaga 2010; Pérez-Figueroa 2013).

Approximately 300 ng of genomic DNA was digested for 3 h at 37 °C in each of two parallel reactions, containing 5 U of *Eco*RI-HF and 5 U of either *Msp*I or *Hpa*II (all New England Biolabs) in a total volume of 10 μL. Ligation adaptors were prepared from single-stranded oligonucleotides (*Eco*RI: 5’–CTCGTAGACTGCGTACC–3’ and 5’–AATTGGTACGCAGTCTAC–3’, Vos *et al.* 1995; *Msp*I/*Hpa*II: 5’–GACGATGAGTCTAGAA–3’ and 5’–CGTTCTAGACTCATC–3’, Xu *et al.* 2000) by mixing equal amounts followed by incubation in a G-Storm GS-1 thermocycler at 95 °C for 5 min and slowly cooling down to room temperature within c. 30 min. A ligation mix containing 5 pmol of *Eco*RI adaptor, 50 pmol of *Msp*I/*Hpa*II adaptor and 1 U of T4 DNA ligase (New England Biolabs) in a volume of 5 μL was added to the digests and incubated at 20 °C overnight. The enzymes were then heat-deactivated at 65 °C for 20 min.

Preselective PCRs were carried out in a total volume of 10 μL containing 1 μL of the digestion/ligation reaction, 0.5 μm each of *Eco*RI+A (5’–GACTGCGTACCAATTCA–3’; Vos *et al.* 1995) and *Msp*I/*Hpa*II+T (5’–GATGAGTCTAGAACGGT–3’; Xu *et al.* 2000) preselective primers, 0.15 U of VELOCITY DNA polymerase (Bioline), 1X VELOCITY HI-FI buffer (containing 2 mm MgCl_2_), and 0.2 mm of each nucleotide. The PCR profile comprised an initial elongation step at 72 °C for 2 min followed by 30 cycles of denaturation at 95 °C for 20 s, annealing at 56 °C for 30 s and elongation at 72 °C for 1 min, and a final elongation step at 60 °C for 2 min.

Eight primer combinations (Table2) were chosen for selective PCR based on the criteria of fragment number, ease of scoring and levels of polymorphism across four test individuals representing the whole study system. Selective PCRs were carried out in a total volume of 20 μL containing 2 μL of preselective PCR product (diluted 1:10), 0.5 μm of each primer, 0.4 U of Taq DNA polymerase (Qiagen), 1X CoralLoad PCR buffer, 2.5 mm MgCl_2_ and 0.2 mm of each nucleotide. The PCR profile comprised an initial denaturation step at 95 °C for 2 min, 13 TouchDown (Don *et al.* 1991) cycles of denaturation at 95 °C for 20 s, annealing decreasing from 65 °C to 56 °C for 30 s and elongation at 72 °C for 1 min, 23 cycles of denaturation at 95 °C for 20 s, annealing at 56 °C for 30 s and elongation at 72 °C for 1 min (increasing by 2 s per cycle), and a final elongation step at 72 °C for 2 min. Two 10 μL aliquots of PCR product were loaded onto two independent 2% agarose–sodium borate gels, electrophoretically separated out for 60 min at 12 V/cm and poststained with Midori Green DNA stain. Band profiles between 125 bp and 600 bp were scored by eye using high-contrast gel photographs.

**Table 2:** Selective primer combinations used in methylation-sensitive AFLP (MSAP), numbers of scored bands, estimated scoring-error rates and consequential classification into methylation-susceptible (MSL) and nonmethylated (NML) loci

*Eco*RI	*Msp*I/*Hpa*II	Bands	Error rate	MSL	NML
GACTGCGTACCAATTC**ACA**	GATGAGTCTAGAACGG**TTGA**	18	0.17	9	9
GACTGCGTACCAATTC**ACA**	GATGAGTCTAGAACGG**TTCA**	17	0.14	12	5
GACTGCGTACCAATTC**ACA**	GATGAGTCTAGAACGG**TTA**	17	0.13	9	8
GACTGCGTACCAATTC**AGA**	GATGAGTCTAGAACGG**TTGA**	13	0.17	5	8
GACTGCGTACCAATTC**AGA**	GATGAGTCTAGAACGG**TGTT**	17	0.12	10	7
GACTGCGTACCAATTC**AGA**	GATGAGTCTAGAACGG**TAC**	18	0.15	9	9
GACTGCGTACCAATTC**ATC**	GATGAGTCTAGAACGG**TAGA**	15	0.17	9	6
GACTGCGTACCAATTC**ATC**	GATGAGTCTAGAACGG**TTCA**	14	0.21	10	4
		129		73	56

### Statistical analysis

Individual band presence or absence states compared between the *Eco*RI-*Msp*I and *Eco*RI-*Hpa*II digests occur in four possible patterns: +/+, –/+, +/– and –/–. In the case of vertebrates, where methylation almost exclusively occurs on the inner cytosine (CpG) of the restriction site (Angers *et al.* 2010; Fulneček & Kovařík 2014), these patterns are interpreted as absence of methylation, hemimethylation (methylation present on one strand only), methylation of the inner cytosine on both strands (hereafter: full methylation) and absence of restriction site, respectively (Xu *et al.* 2000; Pérez-Figueroa 2013). Epigenetic variation can then be assessed from bands with polymorphic methylation states, whereas information on genotypic variation can be extracted from polymorphic bands with methylation states below a particular scoring-error threshold, representing band presence (+/+, –/+ or +/–) or absence (–/–) equivalent to classic AFLP loci (Herrera & Bazaga 2010; Pérez-Figueroa 2013). Total primer combination-specific error thresholds *e*_*T*_ = *e*_*M*_ + *e*_*H*_ − 2*e*_*M*_*e*_*H*_ (Herrera & Bazaga 2010) were estimated from discordant scores in *Msp*I (*e*_*M*_) and *Hpa*II (*e*_*H*_) profiles of 26 individuals that were processed twice from the same DNA extract. Using the r package *msap* (Pérez-Figueroa 2013) in r 3.0.3 (R Core Team 2014), bands with methylation frequencies (–/+ or +/– states) above or below these error thresholds (*e*_*T*_) were then classified as methylation-susceptible (MSL) or nonmethylated (NML) loci, respectively. Information content in MSL and NML was estimated using Shannon's diversity index, and differences were tested for with a Wilcoxon rank-sum test. Independence between MSL and NML information was tested for by applying a Mantel test with 10^5^ randomizations on individual-by-individual MSL and NML distance matrices (Pérez-Figueroa 2013).

Genome-wide levels of full methylation, hemimethylation and absence of methylation were estimated for each individual based on methylation-state frequencies in MSL. Differences in median methylation levels among populations were tested for significance using Kruskal–Wallis tests. Associations of methylation levels with individual parasite load were tested for by Spearman rank correlation. Epigenetic (MSL) and genetic (NML) differentiation among populations was visualized by principal coordinates analysis (PCoA). Using two-level amova with 10^5^ randomizations in the r package *pegas* (Paradis 2010), epigenetic (MSL) and genetic (NML) variances were partitioned into hierarchical components by assigning the populations to three similarly sized groups according to median parasite load (Fig.1). Pairwise epigenetic and genetic differentiation was estimated and tested for using amova-based differentiation statistics (*F*_ST_). Multiple testing was accounted for by calculating false discovery rate (FDR) adjusted *P*-values (= *q*-values) using the r package *fdrtool* (Strimmer 2008). Associations of pairwise differentiation with geographical distances and differences in median parasite load were examined using Mantel tests with 10^5^ randomizations.

Disproportionately differentiated methylation states were identified using *F*_ST_ outlier approaches (Paun *et al.* 2010; Chwedorzewska & Bednarek 2012; Schrey *et al.* 2012). The methylation states of each band were coded as up to three binary variables, each of which represents either the fully methylated, hemimethylated or unmethylated state of the epilocus (‘Mix2’ algorithm in the r script *msap_calc*; Schulz *et al.* 2013). Methylation states with a frequency below 0.05 or above 0.95 and states derived from an NML were removed. Linkage disequilibrium was tested for in genepop 4.2.1 (Raymond & Rousset 1995; Rousset 2008) with 10 000 MCMC dememorizations, 100 batches of 5000 MCMC iterations and a significance threshold of α = 0.05. Tests for *F*_ST_ outliers were carried out using bayescan 2.0 (Foll & Gaggiotti 2008; Pérez-Figueroa *et al.* 2010), running 2·10^6^ iterations (run length 10^5^; thinning interval 20) after 20 pilot runs (10^4^ iterations each) and a burn-in of 5·10^5^, and selecting outliers at *q*≤0.05. For comparison and corroboration, the analysis was repeated using the dfdist algorithm implemented in mcheza (Antao & Beaumont 2011), running 10^5^ simulations with ‘neutral *F*_ST_’ and ‘force mean *F*_ST_’ options, and selecting loci outside the upper tail of the 95% CI.

Associations of binary methylation states with individual parasite load (e.g. Paun *et al.* 2010; Herrera & Bazaga 2011) were examined by fitting multiple generalized estimating equations (GEE) with exchangeable correlation structure within populations using *geepack* (Halekoh *et al.* 2006), thus accounting for within-population correlation of methylation states caused by a shared environment due to kin and population structure (Piertney *et al.* 1998, 1999, 2000). Potential effects of sex (Boks *et al.* 2009; Liu *et al.* 2010), age (Fraga *et al.* 2005; Boks *et al.* 2009) and physiological condition (Fitzpatrick & Wilson 2003; Meaney & Szyf 2005; Franco *et al.* 2008) on methylation were accounted for by including supra-orbital comb area as an additional explanatory variable. Comb area was strongly associated with sex (*t* = 7.23; *P* < 0.001), age (*t* = −2.65; *P* = 0.009) and weight (*t* = 3.59; *P* < 0.001) but not with parasite load (*t* = 1.39; *P* = 0.17). These relationships confirm comb area as a sexual signal and an indicator of physiological condition that is not necessarily reflected in parasite load (Mougeot *et al.* 2004, 2009; Martínez-Padilla *et al.* 2010), but instead in testosterone-dependent immune function, oxidative status or physiological stress (Mougeot & Redpath 2004; Bortolotti *et al.* 2009; Mougeot *et al.* 2010a). Including this single proxy variable rather than a range of interrelated variables reflects the biological relationships of the system and avoids multicollinearity in the model (Graham 2003).

The association analysis was repeated with two other software packages to provide congruence across different model approaches: First, using sam (Joost *et al.* 2008) which implements a logistic regression with a single explanatory variable and ignores any potential correlation among observations. Second, using bayenv2 (Günther & Coop 2013) which tests for association with a single explanatory variable using both a linear model and Spearman rank correlation while accounting for among-population structure through neutral parameterization by control data. Neutral parameterization was performed twice, using either NML as genetic AFLP loci or a set of 260 neutral SNPs (M. A. Wenzel *et al*., unpublished) for comparison and corroboration. bayenv2 was run for 10^6^ iterations both for neutral parameterization and locus testing. Methylation states were considered to be meaningfully associated with parasite load if the regression coefficients (β_1_) of the GEE or sam models were outside the 5% / 95% percentiles of the distribution or if *P* ≦ 0.05. Analogous criteria for the bayenv models were |ρ| ≧ 0.2 or Bayes factor ≧ 2. FDR correction was made for the GEE and sam models, but significance after FDR correction (*q* ≦ 0.1) was taken as additional confidence rather than a strict criterion.

### Functional characterization of epiloci

Those methylation states that were *F*_ST_ outliers or associated with parasite load according to at least one of the three model approaches were pooled, and the corresponding epiloci (MSAP bands) were identified. These bands were then gel-extracted, cloned and Sanger-sequenced to obtain locus identity and physiological functions as a means to ascertain how differential methylation at these loci may take effect.

Bands were picked from the gel using a 10 μL pipette tip, which was then placed in a 0.2 mL PCR tube containing 12 μL of water and incubated at room temperature for 30 min. Upon removal of the tip, 8 μL of PCR mixture was added; the final volume of 20 μL then contained 0.5 μm of each preselective PCR primer, 0.5 U of DNA polymerase (Sigma-Aldrich), 2.5 mm MgCl_2_, 10 mm Tris-HCl, 5 mm KCl, and 0.2 mm of each nucleotide. The PCR profile comprised an initial denaturation step at 95 °C for 2 min, 18 TouchDown (Don *et al.* 1991) cycles of denaturation at 95 °C for 15 s, annealing decreasing from 65 °C to 56 °C for 15 s and elongation at 72 °C for 20 s, 20 cycles of denaturation at 95 °C for 15 s, annealing at 56 °C for 15 s and elongation at 72 °C for 20 s, and a final elongation step at 72 °C for 2 min. PCR products were ligated into Promega pGEM®-T Easy Vector plasmids and transformed into JM109 competent cells according to the manufacturer's instructions. Colonies were screened using standard T7 and SP6 primers in a PCR mixture containing 0.5 μm of each primer, 0.5 U of DNA polymerase (Sigma-Aldrich), 2.5 mm MgCl_2_, 10 mm Tris-HCl, 5 mm KCl and 0.2 mm of each nucleotide. The PCR profile consisted of an initial denaturation step at 95 °C for 2 min followed by 35 cycles of denaturation at 95 °C for 20 s, annealing at 55 °C for 20 s and elongation at 72 °C for 50 s, and a final elongation step at 72 °C for 2 min. Three to four clones per band were Sanger-sequenced on an ABI 3730XL automatic capillary sequencer (Beckman Coulter Genomics, Takeley, UK).

Sequences were aligned and trimmed in geneious 7.1.2 (Drummond *et al.* 2014) and queried against the ncbi RefSeq protein database using blastx (Altschul *et al.* 1997). Additionally, as most MSAP loci are expected to fall outside coding regions (Caballero *et al.* 2013), the clone sequences were mapped to the chicken (*galGal4*) and turkey (*melGal1*) genomes using blat (Kent 2002). The genomic locations of the best hits (highest match score) were then used to identify the nearest ensembl annotations (retrieved from the UCSC table browser; Karolchik *et al.* 2004). Associated gene names, gene descriptions and geneontology terms (The Gene Ontology Consortium 2000) were retrieved from ensembl biomarts (Kinsella *et al.* 2011). Frequencies of each go annotation among the gene sequences were calculated using blast2go (Conesa *et al.* 2005; Conesa & Götz 2008) and custom r scripts.

## Results

Estimated individual parasite loads among the 234 sampled birds ranged from 4 to 9283 worms per bird and population medians ranged from 4 to 2222 worms per bird (Table1). A total of 129 MSAP bands (13–18 per primer combination) were scored in all individuals. Error rates ranged from 0.12 to 0.21, resulting in 73 bands (c. 125–600 bp) to be classified as methylation-susceptible (MSL) and 56 (c. 125–500 bp) as nonmethylated (NML) loci (Table2). Of these, 62 (85%) and 33 (59%) were polymorphic, with marginally different Shannon diversity indices of *I* = 0.44 ± 0.22 SD and *I* = 0.36 ± 0.20 SD, respectively (*W* = 1248; *P* = 0.08). Individual-based epigenetic variation was independent of genetic variation (*r* = 0.02; *P* = 0.23).

**Fig 2:**
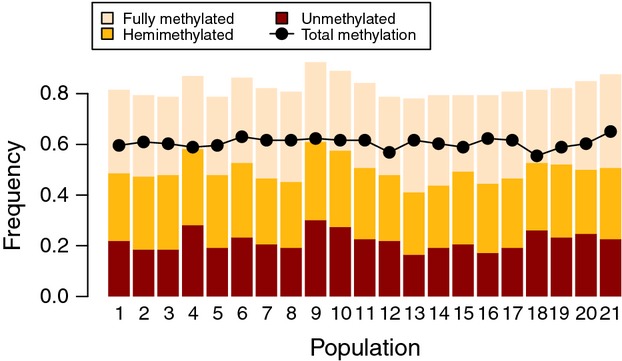
Median genome-wide methylation levels (full methylation, hemimethylation and absence of methylation) per population. Dots represent the summed frequencies of full methylation and hemimethylation.

**Fig 3:**
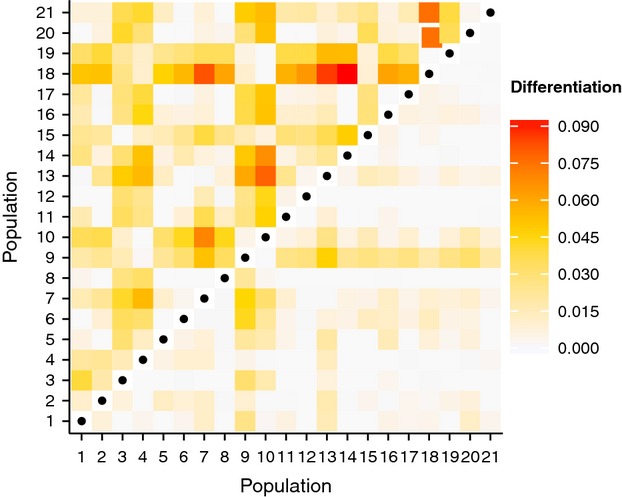
Pairwise epigenetic (above diagonal) and genetic (below diagonal) differentiation (amova-based *F*_ST_) among populations.

The 21 populations varied significantly in median genome-wide full methylation (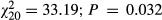), hemimethylation (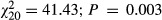) and absence of methylation (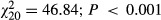), but there was no geographical pattern (Fig.2). Parasite load was weakly positively correlated with genome-wide hemimethylation (ρ = 0.15; *P* = 0.02), but not correlated with full methylation (ρ = −0.10; *P* = 0.11) or absence of methylation (ρ = −0.07; *P* = 0.29). Principal coordinates analysis of epigenetic and genetic variation explained only 4–6% of the total variation and showed substantial overlap among populations (Fig. S1, Supporting information). Nevertheless, global epigenetic differentiation among populations was significant (*F*_SC_ = 0.0227; *P* < 0.001) and considerably stronger than genetic differentiation (*F*_SC_ = 0.0058; *P* < 0.001). Pairwise epigenetic and genetic differentiation (*F*_ST_) ranged from −0.012 to 0.091 (113 of 210 pairs significant at FDR < 0.05) and from −0.012 to 0.048 (16 of 210 pairs significant at FDR < 0.05), respectively (Fig.3). There was no isolation-by-distance pattern in either epigenetic (*r* = 0.02; *P* = 0.38) or genetic (*r* = −0.13; *P* = 0.87) differentiation. Seven populations that were not clustered geographically (locations 3, 4, 9, 10, 15, 18 and 19) showed disproportionate epigenetic differentiation compared with all other populations (Fig.3). Of these, populations 9 and 10 were also disproportionately genetically differentiated, causing a weak correlation between pairwise epigenetic and genetic differentiation matrices (*r* = 0.21; *P* = 0.06). Assigning populations to groups by median parasite load (Fig.1) did not explain any proportion of the molecular variances (Table3). Similarly, pairwise differences in median parasite load did not explain pairwise epigenetic (*r* = −0.08; *P* = 0.75) or genetic (*r* = −0.11; *P* = 0.80) differentiation.

**Table 3:** Two-level amova of methylation-susceptible (MSL) or nonmethylated (NML) MSAP bands among populations grouped by median parasite load (Fig. 1)

	DF	SSD	MSD	Variance	Fixation index
*MSL*					
Among groups	2	0.7597	0.3799	−0.0001 (−0.13%)	*F*_CT_ = −0.0001; *P* = 0.36
Among populations within groups	18	6.7840	0.3769	0.0070 (2.27%)	*F*_SC_ = 0.0227; *P* < 0.001
Within populations	213	63.9168	0.3001	0.3001 (97.74%)	
Total	233	71.4605	0.3067		
*NML*					
Among groups	2	0.5473	0.2737	−0.0004 (−0.13%)	*F*_CT_ = −0.0013; *P* = 0.99
Among populations within groups	18	5.4284	0.3016	0.0016 (0.58%)	*F*_SC_ = 0.0058; *P* < 0.001
Within populations	213	60.3803	0.2835	0.2835 (99.56%)	
Total	233	66.3560	0.2848		

**Fig 4:**
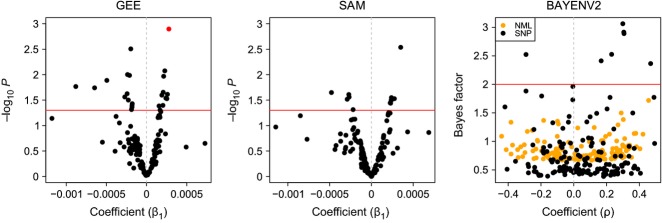
Coefficients and statistical significance of regression tests between epilocus-specific methylation states and parasite load using generalized estimating equations (GEE), logistic regression (sam) and Bayesian environmental analysis (bayenv2 with neutral parameterization by either NML or SNP genetic data). Each dot represents one methylation state. The red lines indicate significance thresholds (*P* ≦ 0.05 or Bayes factor ≧ 2), and red dots represent models with FDR-corrected *q* ≦ 0.1.

In contrast, epilocus-by-epilocus analyses revealed associations of locus-specific methylation states with parasite load. The 62 polymorphic MSL were transformed into 132 binary methylation states with no evidence of linkage disequilibrium among them. bayescan highlighted eight *F*_ST_ outlier methylation states, all of which were corroborated in mcheza. Overall, 35 individual methylation states (9 fully methylated, 12 hemimethylated and 14 unmethylated states), representing 25 epiloci, were significantly associated with parasite load using at least one model approach (GEE, sam, bayenv2). Of these, 19 methylation states were positively associated with parasite load and 16 states negatively. In the cases of ten epiloci, two methylation states were associated with parasite load and the unmethylated state always had the opposite directionality of association of the methylated state. Two *F*_ST_ outliers were not associated with parasite load. Regression results of each analysis method are summarized as volcano plots (Fig.4). *F*_ST_ outliers and significantly associated methylation states are organized by corresponding epiloci and statistical support based on congruence across analysis approaches (Table4).

**Table 4:** Epilocus-specific methylation states significantly associated with parasite load or identified as *F*_ST_ outliers. Each epilocus is listed with size, type of methylation state (U = unmethylated, H = hemimethylated, M = fully methylated), sign of regression coefficient (β_1_ for GEE/sam and ρ for bayenv2) and strength of statistical support. Results for bayenv2 are given for separate analyses using either nonmethylated MSAP loci (NML) or a set of 260 neutral SNPs for neutral parameterization

Epilocus (MSAP band)								bayenv2^b^			
ID	Name	Size	State ID	Type	Coefficient	GEE^a^	sam^a^	NML	SNP	bayescan^c^	mcheza^d^
1	MSAP_4.10_01	500	ML201	U	–	**	**	2		***	***
			ML234	H	+		**			***	***
2	MSAP_3.12_05	400	ML189	H	+				2**	***	***
			ML245	U	–					***	***
3	MSAP_3.12_04	450	ML192	M	+			2		***	***
			ML244	U	–			2		***	***
4	MSAP_2.17_02	475	ML238	M	–					***	***
			ML117	U	+	**		3	4		
5	MSAP_2.13_10	275	ML159	M	+			3	2		***
6	MSAP_2.13_03	500	ML224	H	–			3	2**		***
			ML220	U	+			3	3***		
7	MSAP_2.6_09	300	ML202	H	+	***	**	3	2**	***	**
			ML218	U	–	**	**	3	2		**
8	MSAP_3.16_01	600	ML209	H	+	**	**	3	2		**
			ML128	U	–	**	*				
9	MSAP_4.13_04	450	ML207	H	+	**		3			*
10	MSAP_4.10_15	125	ML98	U	–	**	*				
			ML194	M	+	**	**	2			
11	MSAP_3.16_17	130	ML172	U	–	**	**				
12	MSAP_3.12_01	600	ML204	H	+	**	**	4	3**		
			ML210	U	–			3	2		
13	MSAP_4.13_08	250	ML213	H	+	**	**	2	2		
14	MSAP_2.17_14	160	ML228	U	–	**	**				
15	MSAP_3.16_07	320	ML272	M	–	**	**	2	2		
16	MSAP_4.10_05	390	ML143	U	+	**		2	3		
17	MSAP_3.6_09	225	ML100	M	–	*	*				
18	MSAP_4.13_06	390	ML284	H	+	*	*	4	3		
19	MSAP_3.12_09	320	ML288	M	+	*	*				
20	MSAP_4.13_01	600	ML99	U	–	*	*				
21	MSAP_4.13_11	150	ML275	H	+		*				
22	MSAP_3.12_02	550	ML105	M	+			4	4**		
23	MSAP_2.6_01	600	ML107	H	+			4	4*		
24	MSAP_3.16_03	500	ML153	U	–			2			
			ML262	H	+			2	2**		
25	MSAP_3.16_13	200	ML281	M	–			3	3		

a *: absolute value of coefficient outside 5% / 95% percentiles; **: *P* ≦ 0.05; ***: *q* ≦ 0.1.

b 2: |ρ| ≧ 0.2; 3: |ρ| ≧ 0.3; 4: |ρ| ≧ 0.4; **: Bayes factor ≧ 2; ***: Bayes factor ≧ 3.

c ***: *q* ≦ 0.05.

d *: *P* ≧ 0.90; **: *P* ≧ 0.95; ***: *P* ≧ 0.99.

**Fig 5:**
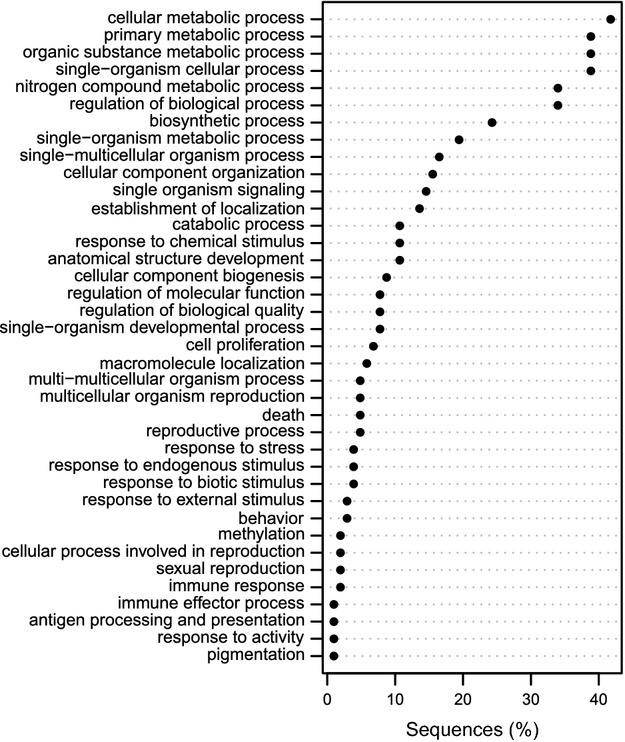
Frequencies of level-3 ‘biological process’ geneontology (go) annotations of genes near MSAP band clone sequences mapped to chicken and turkey genomes.

Sanger sequencing of cloned epiloci yielded one to four sequences per epilocus (77 unique sequences, available from genbank Accession nos KJ655444–KJ655520), suggesting some incidence of clone band size homoplasy. Of these, only six (representing five MSAP bands) provided characterized RefSeq protein hits with an expected value below 1. At least one sequence per band could be mapped to the chicken or turkey genomes and most sequences were mapped to noncoding regions. The distance between the midpoints of the mapped sequence and the nearest ensembl gene annotation ranged from 96 bp to 630 Kbp (c. 0.003 mM to 1.764 cM; Andreescu *et al.* 2007). The geneontology annotations of these protein hits or nearest annotated genes included numerous biological process categories (Fig.5), most notably immune system, epigenetic mechanisms, cell cycle/proliferation and energy metabolism (Table5). Immune system genes included B-cell (PRDM1/BLIMP1, IKZF3) and T-cell (EOMES) proliferation regulators, MHC binding proteins (MARCH1) and enzymes involved in somatic hypermutation (DNTT). Intriguing additional findings included an rRNA methyltransferase (TFB2M), a histone acetyltransferase (KAT2B), two microRNAs (MIR1575, MIRLET7G), one small nucleolar RNA (SNORD111) and a transposable element (POGK). Complete characterizations for every sequence including full geneontology terms are presented in Table S1 (Supporting information).

**Table 5:** Sequence characterization of select MSAP bands detailing gene names, descriptions, key geneontology terms and accessions of either blastx top hit (with expected E-value) or nearest ensembl annotation (with distance in base pairs) in chicken (ENSGAL) or turkey (ENSMGA) genomes

Band ID	Gene name	Description	Accession	E-value	Distance	Key go terms
		*Immune system*				
12	PRDM1/BLIMP1	PR domain containing 1, with ZNF domain	ENSGALT00000024824		6.28E+03	positive regulation of B-cell differentiation (GO:0045579); negative regulation of B-cell proliferation (GO:0030889)
17	EOMES	eomesodermin	ENSGALT00000042658		7.56E+04	CD8-positive, alpha-beta T-cell differentiation involved in immune response (GO:0002302)
5	IKZF3	IKAROS family zinc finger 3 (Aiolos)	ENSMGAT00000003138		6.74E+03	regulation of B-cell differentiation (GO:0045577)
10, 11, 14	MARCH1	membrane-associated ring finger (C3HC4) 1, E3 ubiquitin protein ligase	ENSMGAT00000002357		7.50E+04	MHC protein binding (GO:0042287); immune response (GO:0006955)
22	DNTT	DNA nucleotidylexotransferase	ENSGALT00000033285		6.63E+04	DNA binding (GO:0003677)
		*Epigenetic mechanisms*				
17	TFB2M	mitochondrial 12S rRNA dimethylase 2	ENSGALT00000017328		1.55E+04	rRNA methylation (GO:0031167)
8	KAT2B	histone acetyltransferase KAT2B isoform X2	XP_426001.4	4.06E-08		lysine N-acetyltransferase activity (GO:0004468)
15	MIR1575	gga-mir-1575	ENSGALT00000042383		4.85E+04	N/A (microRNA)
10	MIRLET7G	gga-let-7g	ENSGALT00000028969		9.60E+01	N/A (microRNA)
23	SNORD111	small nucleolar RNA SNORD111	ENSGALT00000042232		2.48E+02	N/A
		*Cell cycle and proliferation*				
22, 3	PHACTR3	phosphatase and actin regulator 3 isoform X3	XP_004947133.1	3.90E-37		actin binding (GO:0003779)
			ENSGALT00000007780		1.43E+04	
25	DIAPH3	diaphanous-related formin 3	ENSMGAT00000016825		3.90E+04	cellular component organization (GO:0016043)
8	TUC338	transcribed ultra-conserved region 338	ENSGALT00000044640		4.95E+05	N/A (hepatocyte proliferation)
8	WDR26	WD repeat domain 26	ENSGALT00000015154		9.62E+02	protein binding (GO:0005515)
23	SF3B3	splicing factor 3B subunit 3, partial	XP_005009141.1	1.31E-33		mRNA processing (GO:0006397)
24	POGK	pogo transposable element with KRAB domain	ENSMGAT00000013461		8.00E+04	regulation of transcription, DNA-templated (GO:0006355)
		*Energy metabolism*				
20	SDHA	succinate dehydrogenase [ubiquinone] flavoprotein subunit A	XP_005419422.1	1.99E-04		mitochondrial respiratory chain complex II (GO:0005749)
19	LARGE	like-glycosyltransferase	ENSMGAT00000014370		2.58E+05	glycoprotein biosynthetic process (GO:0009101)
18	PPARGC1A	peroxisome proliferator-activated receptor gamma, coactivator 1 alpha	ENSGALT00000023263		1.32E+05	positive regulation of cellular respiration (GO:1901857)
22	ACOX2	acyl-CoA oxidase 2, branched chain	ENSGALT00000011555		4.72E+03	fatty acid metabolic process (GO:0006631)

## Discussion

We highlight significant fine-scale epigenetic structure among red grouse populations in north-east Scotland and associations of parasite load with methylation patterns on a locus-specific, but not genome-wide level. Some parasite-associated epiloci were mapped to genomic regions close to immune genes or genes for epigenetic factors such as histone acetyltransferases and microRNAs, providing intriguing correlational evidence for epigenetically regulated host–parasite interactions in a wild bird species.

The sampled grouse populations differed significantly in genome-wide methylation levels and were significantly epigenetically differentiated, providing first evidence of significant epigenetic differentiation among wild bird populations on a small geographical scale. This is in surprising contrast with introduced house sparrow populations in Kenya and Florida, which are not epigenetically differentiated within Kenya or even across continents, possibly due to high epigenetic diversity compensating for low genetic diversity and inbreeding (Schrey *et al.* 2012; Liebl *et al.* 2013). The magnitude of epigenetic differentiation observed among grouse was considerably stronger than genetic differentiation derived from nonmethylated MSAP loci. Similarly, epigenetic diversity was also marginally greater than genetic diversity. There was no evidence for an association of epigenetic with genetic variation, suggesting autonomy of the captured epigenetic variation (Richards 2006). In concert, these findings suggest that DNA methylation may be an important source of ecologically relevant variation in these grouse populations (Lira-Medeiros *et al.* 2010; Richards *et al.* 2012).

Epigenetic differentiation among populations could be caused by neutral epimutations subject to random drift as a consequence of limited dispersal (Massicotte *et al.* 2011). Significant epigenetic differentiation (*F*_ST_ = 0.3) consistent with such short-distance dispersal has been reported among great roundleaf bat populations in China (Liu *et al.* 2012). However, this scenario is unlikely to be the case in grouse, because epigenetic differentiation was not attributable to isolation by distance, despite genetic evidence for population structure from microsatellite data (Piertney *et al.* 1998, 1999). A more likely explanation for the observed epigenetic differentiation may be environmental heterogeneity across the landscape, such as differential parasite load. Considerable epigenetic differentiation (*F*_ST_ = 0.1–0.3) is commonly found among plant populations in contrasting environments on similar spatial scales as our study (Herrera & Bazaga 2010; Lira-Medeiros *et al.* 2010; Herrera *et al.* 2013; Schulz *et al.* 2013). Notably, Chwedorzewska & Bednarek (2012) found marked epigenetic differentiation (*F*_ST_ = 0.5) among Polish and Antarctic annual bluegrass populations, and Richards *et al*. (2012) report strong epigenetic differentiation (*F*_ST_ = 0.5–0.8) of Japanese knotweed populations during invasion into different environments. However, the observed genome-wide epigenetic patterns among grouse populations were not attributable to parasite load. Instead, these patterns were predominantly driven by the disproportionate differentiation of seven populations that were neither geographically clustered nor similar in parasite load. Intriguingly, two of these populations were both epigenetically and genetically disproportionately differentiated. These patterns may be caused by demographic or adaptive processes due to environmental factors that were not considered in this study, but warrant further investigation.

Our main objective was to ascertain whether parasite load is linked to epigenetic variation in wild grouse populations. Neither population-based genome-wide epigenetic differentiation nor individual-based genomewide methylation levels were associated with parasite load, apart from a weak positive association with genome-wide hemimethylation. However, epilocus-by-epilocus analyses revealed associations of methylation states at particular epiloci with parasite load and also disproportionate differentiation (*F*_ST_ outliers). This is no contradiction because epilocus-specific associations with parasite load can be either positive or negative, which precludes the detection of association when methylation levels are averaged across loci to provide genome-wide estimates (Paun *et al.* 2010; Schrey *et al.* 2012). Indeed, environmental factors may well impact a finite number of individual epiloci rather than genome-wide methylation, which becomes manifested in differentiation or association at specific epiloci (Paun *et al.* 2010; Chwedorzewska & Bednarek 2012) even in the absence of genome-wide differentiation (Schrey *et al.* 2012). The observed *F*_ST_ outliers suggest such an impact by unknown environmental factors, because not all outliers were also associated with parasite load. Nevertheless, our findings vividly demonstrate a locus-specific relationship between epigenetic variation and a biotic environmental stressor.

Given that controlled transcriptomic experiments have previously demonstrated that parasite infection alters gene expression in liver, spleen and caecum tissues in red grouse (Webster *et al.* 2011a, b), one possible interpretation of epilocus-specific association with parasite load is that parasites cause epilocus-specific methylation changes that impact gene expression (Angers *et al.* 2010; Paun *et al.* 2010; Jones 2012). Similarly to transcriptomic changes, such parasite-driven methylation changes would then present a transient response to an environmental factor during the bird's lifetime without assuming inheritance of methylation states (Skinner 2011). Among our association results, methylation was predominantly positively associated with parasite load (76%) and absence of methylation negatively (79%), often consistently in complement at the same epilocus. This suggests a predominant pattern of methylation-mediated positive association of parasite load with down-regulation of gene expression (Angers *et al.* 2010; Jones 2012). The rarer inverted observation that methylation was negatively associated with parasite load and absence of methylation positively suggests that parasite infection may also cause demethylation at some loci and concomitant up-regulation of gene expression (Angers *et al.* 2010; Jones 2012). The physiological processes highlighted by the geneontology terms of the sequenced epiloci were manifold, corroborating the view that parasite infection impacts physiological condition through a wide range of vital cellular processes rather than single categories such as the immune system (Hill 2011; Webster *et al.* 2011a, b). Immune system processes were only a small subset, yet methylation at all but one of those immune genes was positively associated with parasite load, consistent with immunosuppressive effects of helminth infection (Maizels & Yazdanbakhsh 2003; Biron & Loxdale 2013). Most intriguingly, some epiloci were linked to genes that are themselves involved in epigenetic mechanisms, including rRNA methylation, histone acetylation and RNA interference by small RNAs. These mechanisms are primarily involved in regulating ribosomal translation and chromatin remodelling (He & Hannon 2004; Shahbazian & Grunstein 2007; Metodiev *et al.* 2009; Bannister & Kouzarides 2011). In consequence, parasite-linked changes in methylation patterns at these loci may regulate the expression of epigenetic factors that regulate gene expression or chromatin remodelling elsewhere in the genome, providing an enticing, yet speculative perspective on the consequences of environmentally induced epigenetic states (Feil & Fraga 2012).

An alternative functional interpretation of methylation changes is facilitation of nucleotide sequence mutations rather than regulation of gene expression. Methylated cytosine is substantially more liable to deamination than unmethylated cytosine (Lutsenko & Bhagwat 1999; Poole *et al.* 2003), suggesting that increased locus-specific methylation following parasite infection may create mutational hot spots in gene bodies that could provide genetic variation during, for example, somatic hypermutation in immune genes of proliferating hepatic cells (Racanelli & Rehermann 2006; Jones 2012). Another important function of methylation is the silencing of transposable elements that become released following demethylation (Suzuki & Bird 2008; Zemach *et al.* 2010; Jones 2012). The few observed cases of association of absence of methylation with parasite load could therefore be explained as a release of transposable elements that could create somatic genetic variation to facilitate systemic responses to parasite infection. This interpretation would be consistent with the frequently observed phenomenon that demethylation increases phenotypic variance (Bossdorf *et al.* 2010; Vergeer *et al.* 2012). One epilocus was indeed mapped to the vicinity of a transposable element, but its association with parasite-linked hemimethylation would impede transposition at high parasite load rather than induce it, suggesting this may be coincidental.

These functional interpretations of parasite-associated methylation have to remain speculative because no independent genomic data are available for red grouse. However, our finding that most epiloci were mapped to noncoding sequence regions is consistent with gene regulation either directly through methylation changes in the CpG islands of gene promoters (Angers *et al.* 2010; Jones 2012; Duncan *et al.* 2014) or indirectly through methylation-associated recruitment of complexes that remodel chromatin (Jaenisch & Bird 2003; Bannister & Kouzarides 2011; Feil & Fraga 2012). Given that many epiloci were mapped to the vicinity of a gene, these genes may be directly affected by these epiloci, but this becomes increasingly difficult to reconcile with increasing genomic distances. Although long-range transcriptional regulation exists (Kleinjan & van Heyningen 2005), it is likely that many of those epiloci in noncoding regions are not specifically involved in regulating the genes in their vicinity, but may instead be involved in remodelling chromating with potentially far-reaching regulatory consequences (Jaenisch & Bird 2003; Bannister & Kouzarides 2011; Feil & Fraga 2012). Clearly, functional genomics analyses in the context of a controlled infection experiment would be required to establish causal links between methylation changes and their genomic and physiological consequences (Duncan *et al.* 2014). Nevertheless, despite their speculative nature, our interpretations describe a number of hypothesis-generating mechanisms that may direct further exciting research.

The rationale of our study was to detect a correlational epigenetic signature of parasite load in red grouse populations that could be intepreted as a transient epigenetic response to parasites, similarly to a transcriptomic response (Webster *et al.* 2011b). This was prompted by a large body of red grouse research that has identified parasite infection as an important effect on physiology and behaviour (e.g. Fox & Hudson 2001; Mougeot *et al.* 2005a, 2010a, Mougeot *et al.* 2010a,b; Martínez-Padilla *et al.* 2007; Vergara *et al.* 2012). From this point of view, our results provide evidence for a broad epigenetically mediated physiological response to parasites and suggest that helminths may effect manipulations of host physiology and behaviour at least partially through transient epigenetic mechanisms (Maizels & Yazdanbakhsh 2003; Biron & Loxdale 2013; Poulin 2013). The evolutionary relevance of these mechanisms is difficult to ascertain (Richards *et al.* 2010; Duncan *et al.* 2014). Vertical transmission of methylation patterns in an analogous way to genetic polymorphisms exists (Jablonka & Raz 2009; Petronis 2010; Skinner 2011; Smith & Ritchie 2013; D’Urso & Brickner 2014), particularly in plants (Salmon *et al.* 2008; Verhoeven *et al.* 2010; Herrera *et al.*, 2013, 2014), but the dearth of transgenerational ecological epigenetics studies on animals leaves a large scope for exciting future research. If inheritance of methylation patterns could be demonstrated in red grouse, parasite load might potentially be a consequence of methylation changes rather than a cause. Inherited methylation patterns may then contribute to an innate resistance to parasites (‘condition’) without necessarily undergoing alterations as a consequence of infection themselves (Poulin & Thomas 2008).

In conclusion, our study highlights the potential for ecological epigenetics to illuminate mechanisms of plasticity and adaptation in the context of host–parasite interactions in natural systems. We also highlight the necessity of independent transcriptomics and genomics data to overcome conceptual difficulties in interpreting epigenetics patterns. In spite of these challenges, DNA methylation may be key to understanding the generation of phenotypic variation and the evolution of complex phenotypes in the absence of genetic variation, indicating that the study of epigenetic causes and consequences of environmentally induced phenotypes may be paramount to understanding how plasticity-conferred functional variation may contribute to eco-evolutionary processes (Bossdorf *et al.* 2008, 2010; Petronis 2010; Roux *et al.* 2011).

## Data accessibility

DNA clone sequences: genbank Accession nos KJ655444–KJ655520.

Clone sequence characterization including geneontology terms uploaded as supplementary information (Table S1).

Custom R scripts with input files: Dryad 10.5061/dryad.f727j

## Supporting Information

Fig S1 Principal coordinate analysis plots of epigenetic and genetic variation in the study populations.

Table S1 Clone sequence characterization following genome mapping and geneontology annotation. Contains chicken/turkey chromosome locations of mapped clone sequences, nearest ensemble gene identifiers and associated geneontology terms.
