# Supplementary material for: Fine-scale population epigenetic structure in relation to gastrointestinal parasite load in red grouse (Lagopus lagopus scotica)
Source: Mol Ecol. 2014 Jul 24;23(17):4256–73. doi: 10.1111/mec.12833 (PMC4282444; doi:10.1111/mec.12833)
Supplement: Fig S1 — Principal coordinate analysis plots of epigenetic and genetic variation in the study populations. [file mec0023-4256-SD1.pdf]

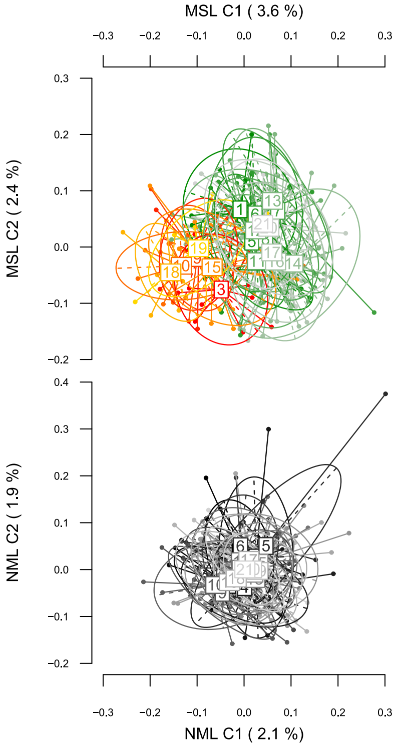

Figure S1
Principal coordinate analysis plots of epigenetic and genetic variation in methylation-susceptible (MSL; top) and non-methylated loci (NML; bottom). Population labels refer to sampling site ID (see main article).
